# Diversity of Myxobacteria—We Only See the Tip of the Iceberg

**DOI:** 10.3390/microorganisms6030084

**Published:** 2018-08-11

**Authors:** Kathrin I. Mohr

**Affiliations:** Microbial Drugs (MWIS), Helmholtz Centre for Infection Research (HZI), 38124 Braunschweig, Germany; kathrin.mohr@helmholtz-hzi.de; Tel.: +49-531-6181-4251

**Keywords:** myxobacteria, diversity, uncultured, secondary metabolites, new antibiotics

## Abstract

The discovery of new antibiotics is mandatory with regard to the increasing number of resistant pathogens. One approach is the search for new antibiotic producers in nature. Among actinomycetes, *Bacillus* species, and fungi, myxobacteria have been a rich source for bioactive secondary metabolites for decades. To date, about 600 substances could be described, many of them with antibacterial, antifungal, or cytostatic activity. But, recent cultivation-independent studies on marine, terrestrial, or uncommon habitats unequivocally demonstrate that the number of uncultured myxobacteria is much higher than would be expected from the number of cultivated strains. Although several highly promising myxobacterial taxa have been identified recently, this so-called Great Plate Count Anomaly must be overcome to get broader access to new secondary metabolite producers. In the last years it turned out that especially new species, genera, and families of myxobacteria are promising sources for new bioactive metabolites. Therefore, the cultivation of the hitherto uncultivable ones is our biggest challenge.

## 1. Introduction

We know little about the real diversity of myxobacteria in the environment. How many myxobacteria are cultivable under standard laboratory conditions? How many resist these cultivation efforts and lie undiscovered in the ground? After an introduction to myxobacteria, the status of antibiotics, myxobacterial secondary metabolites, the Great Plate Count Anomaly phenomenon, and microbial biogeography, the diversity of cultivable and uncultivated myxobacteria in different habitats is presented. Therefore, numerous sequences from the NCBI database were analysed. The intent of this review is to draw attention to the high amount of undiscovered myxobacteria and encourage further discovery and isolation of these hidden treasures with regard to their potential as new antibiotic producers.

## 2. Biology and Phylogeny of Myxobacteria

**Figure 1:**
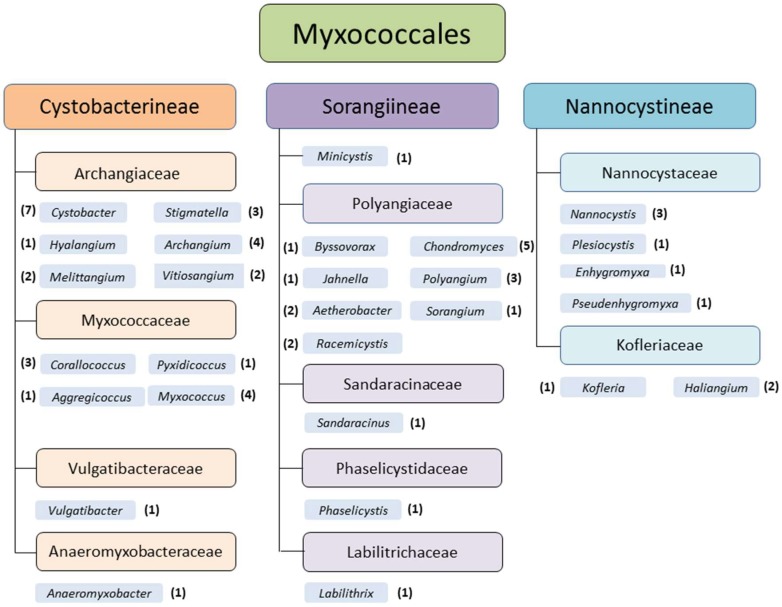
Monophyletic order Myxococcales (delta-proteobacteria), suborders, families, and genera of myxobacteria (status May 2018). The number of species within the genera is mentioned in brackets (original graphic from Corinna Wolf, modified by K. I. Mohr).

Myxobacteria are soil dwelling deltaproteobacteria and are distributed all over the world. Temperate zones, tropical rain forests, arctic tundra, deserts, acidic soils [1,2,3], marine and other saline environments [4,5,6,7], and even caves [8], for example, are appropriate habitats. Myxobacteria can be isolated from various natural sources as soil, bark, rotting wood, leaves of trees, compost [9], or dung of herbivores [1,10]. They live aerobically, except the only described facultative anaerobic genus and species, *Anaeromyxobacter dehalogenans* [11]. Nevertheless, it is highly likely that further facultative or even strictly anaerobic myxobacteria exist, which hitherto withstand the common isolation efforts. Currently the monophyletic order Myxococcales comprises 3 suborders, 10 families, 29 genera, and 58 species (Figure 1).

Myxobacteria are fascinating because of their extraordinary social lifestyle, which is unique in the bacterial domain. Under appropriate environmental conditions, vegetative cells move in swarms by gliding over solid surfaces [12]. Myxobacteria do not have flagella, but two motility systems, used for locomotion, and well studied in *Myxococcus xanthus*, are known: social (S-) motility, powered by retraction of type IV pili is responsible for the movement of cells which travel in groups [13]. In addition, extracellular matrix polysaccharide (EPS), also referred to as fibrils, are used. Therefore, the intrinsic polarity of rod-shaped cells lays the foundation, and each cell uses two polar engines for gliding on surfaces. It sprouts retractile type IV pili from the leading cell pole and secretes capsular polysaccharide through nozzles from the trailing pole [14]. On the other hand, slime secretion enables cell movements, when cells were isolated from the group (adventurous A-motility) [15]. For a detailed description of myxobacterial gliding mechanisms see Nan et al. [13] and Faure et al. [16].

Due to their nutritional behavior and based on their specialization in degradation of biomacromolecules, members of the order Myxococcales can be divided into two groups: predators (the majority), which are able to lyse whole living cells of other microorganisms by exhausting lytic enzymes, and cellulose-decomposers, the latter are represented by the genera *Sorangium* and *Byssovorax* [12]. But, as mentioned for the (facultative) anaerobic myxobacteria, it is also highly likely that further cellulose-degrading genera exist, which successfully resisted standard cultivation attempts.

**Figure 2:**
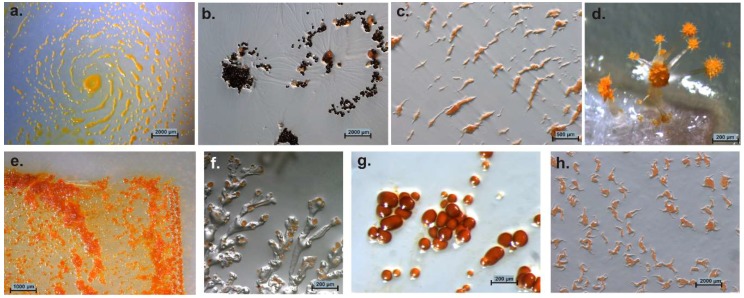
Variation of myxobacterial fruiting bodies. Genus/species, strain designation, (agar medium) are mentioned. (**a**) *Myxococcus xanthus* Mxx42 (P); (**b**) *Cystobacter ferrugineus* Cbfe48 (VY/2); (**c**) *Archangium* sp. Ar7747 (VY/2); (**d**) *Chondromyces* sp. (Stan 21 with filter); (**e**) *Sorangium* sp. Soce 1462 degrading filter paper on Stan 21 agar; (**f**) *Polyangium* sp. Pl3323 (VY/2); (**g**) *Cystobacter fuscus* Cbf18 (VY/2); (**h**) *Corallococcus coralloides* Ccc379 (VY/2).

If nutrients become rare, the cells undergo an impressive process of cooperative morphogenesis. Cells agglomerate and form species-specific fruiting bodies by directed cell movement [12]. These fruiting bodies consist of one to several sporangioles [1]. The architecture of these fruiting bodies ranges from simple, single sporangioles (*M. xanthus*, *Cystobacter* spp.), stalked sporangioles (*M. stipitatus*), or even delicate tree-like structures of high complexity (*Chondromyces* spp.; *Stigmatella* spp.) [10]. Colors of cells/fruiting bodies vary from milky, yellow, orange, red, brown to even black (Figure 2) [1].

A known function of these mainly carotenoid or melanoid pigments is to provide protection against photo-oxidation [17]. Within the fruiting bodies, most of the vegetative cells die and serve as food for the remaining cells, which convert into short and hardy myxospores, especially resistant to desiccation [18,19]. These spores are not as heat-resistant as *Bacillus* spores, but they can survive in the environment and are able to germinate under appropriate conditions even after decades of resting [1]. Therefore, it is possible to isolate myxobacteria from dried environmental samples, which were stored for several years at room temperature [10]. A fruiting body consists of 10^5^–10^6^ cells [18]. This ensures that the new cycle starts with a sufficient amount of cells, necessary for the typical collaborative feeding [20]. A further very interesting feature of myxobacteria is their ability to produce a large number and variety of secondary metabolites, as described in the next section. 

In 1892, Thaxter was the first who described myxobacteria in literature [19]. He found out that *Chondromyces crocatus* was a bacterium and he had discovered its unicellular vegetative stage. This was spectacular, because until such time, *C. crocatus* had been considered a slime mold for more than 20 years [14]. Studies by Bauer [21], Kofler [20], Jahn [22,23], and Kühlwein [24] followed in the early 20th century. Myxobacteria have always fascinated scientists due to their social behavior, including cooperative swarming, group predation, and multicellular fruiting body formation. *Myxococcus xanthus* for example has become one of the model systems for the study of prokaryotic development [25]. Today, beside their capabilities to produce promising bioactive secondary metabolites, myxobacteria are of utmost importance in elucidating multicellular behavior in bacteria, as well as working out social evolution theory.

## 3. Current Status of Antibiotics and Myxobacterial Secondary Metabolites

Before the first antibiotics were commercially available in the early 20th century, people were delivered helplessly to various kinds of infections like pest, cholera, and tuberculosis, which often reached epidemic proportions and have cost the lives of millions of people [26]. In 1940, quinine was used against malaria, the arsenic derivative arsphenamine, Salvarsan, was used against syphilis, and sulfa drugs like Prontosil were used against mainly Gram-positive cocci infections. However, most agents of infectious diseases were still untreatable. The situation improved radically with the detection of the first beta-lactam antibiotic, penicillin, produced by the mold *Penicillium rubens* [27]. Henceforth, soil organisms like fungi [28] and bacteria [29] as producers of secondary metabolites with bioactive properties moved into the focus of research. The Golden Age of Antibiotics started. Aminoglycosides [30], tetracyclines [31], and macrolides [32] are only some examples of important antibiotic classes, discovered in those days. Numerous pharmaceutical companies participated on large-scale screening activities of antibiotic producing organisms, mainly actinobacteria [33]. However, in most cases, it took only a few years from the launch of a new antibiotic to the detection of the first resistant germs [34]. Incorrect use in human medicine, incorrectly prescribed antibiotics, extensive agricultural use and fast spread of resistant bacteria caused by increasing mobility led to substantial problems with multi-drug resistant bacteria. Some of the most problematic germs belong to the so-called ESKAPE-panel: *Enterococcus faecium*, *Staphylococcus aureus*, *Klebsiella pneumoniae*, *Acinetobacter baumannii*, *Pseudomonas aeruginosa*, and *Enterobacter* spp, are mainly responsible for nosocomial infections. Since the 1960s more and more companies retracted from the time- and cost-consuming screening procedures. Of the 18 largest pharmaceutical companies, 15 abandoned the antibiotic field [35]. Indeed, from the late 1960s through the early 1980s, the pharmaceutical industry introduced many new antibiotics to solve the resistance problem. After that the antibiotic pipeline began to dry up and fewer new drugs were brought to market [36]. This led to a dangerous bottleneck of currently available reserve-antibiotics and a widely held concern over the lack of innovation and productivity in the research and development of novel bioactive substances [37]. Eligible countermeasures include the development of synthetic and semi-synthetic drugs, evaluation of rediscovered drugs and the classical screen of natural secondary metabolite producers. Here, especially new genera and species are of great interest [38]. But for natural production of secondary metabolites in large-scale fermentation processes the corresponding producer strains have to be isolated from nature. Maintenance, cultivation, and upscale are challenging. Beside the appropriate expertise and equipment for fermentation and isolation of substances from the fermenter broth, for every producer strain the specific biotic and abiotic conditions need to be determined. Myxobacteria for instance are one of the most promising natural product producers, but demanding with regard to isolation and large-scale cultivation. Successful handling of these organisms places special challenges to microbiologists and biotechnologists in equal measure.

**Figure 3:**
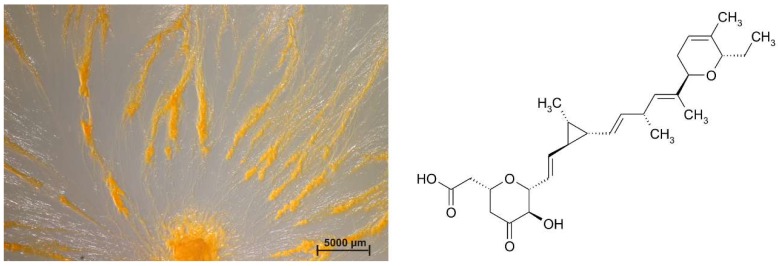
*Sorangium* sp. strain Soce 1014, an ambruticin-producer, swarming on VY/2-agar and the structure of ambruticin A, the first secondary metabolite which was isolated and described from myxobacteria.

Myxobacteria are among the best natural product producers, together with actinomycetes [39], *Bacillus* species [40], and fungi [31]. Even shortly after their discovery, scientists described predatory and cellulolytic action of myxobacteria. Already in 1947, Singh complained that many antibiotics were isolated from various groups of microorganisms, except myxobacteria [41]. He observed that some species of the Myxococcaceae lyse living bacteria, including Gram-negatives such as *Pseudomonas fluorescens* and *Bacterium (Escherichia*) *coli,* and concluded that a detailed study of myxobacteria may be profitable in discovering new antibiotics. In 1955, Mathews and Dudani investigated the lysis of human pathogenic bacteria by myxobacteria [42] and in 1962, Noren and Raper described the antibiotic activity of myxobacteria in relation to their bacteriolytic capacity [43]. But, it took another 15 years until the first antifungal metabolite, ambruticin, was isolated from a *Sorangium* strain (Figure 3) [44].

The majority of myxobacterial compounds are polyketides, non-ribosomal polypeptides, and their hybrids, terpenoids, phenyl-propanoids, and alkaloids [45]. Many of these substances show promising activities against bacteria [46,47], viruses [48], fungi [49], cancer cells [50] immune cells [51], and malaria [52], respectively, as well as unusual modes of action [53]. Many strains produce metabolites belonging to multiple structural classes, as well as a number of chemical variants on each basic scaffold [48]. Whole-genome sequencing of several myxobacterial strains like *Sorangium cellulosum* [54] and *Myxococcus xanthus* [55] has revealed that the secondary metabolite potential is far greater than that suggested by fermentation under standard laboratory conditions.

It is, of course, possible to isolate new substances from known (myxobacterial) species [51,52]. But again: the low hanging fruit have long been harvested and it is more likely to find new substances in new families, genera and species [53,56,57,58,59,60,61]. The study of Hoffmann et al. confirmed this [41]. The authors found a correlation between taxonomic distance and the production of distinct secondary metabolite families, and supported the idea that the chances of discovering novel metabolites are greater by examining strains from new genera rather than additional representatives within the same genus. For comprehensive overviews about secondary metabolites produced by myxobacteria and their mode of action, I recommend Weissman and Müller [48] and Herrmann et al. [49].

## 4. The Great Plate Count Anomaly and Microbial Biogeography

Based on cultivation, approximately only 1% of the naturally occurring bacterial community is known and characterized so far [62]. Most bacterial groups remain uncultured and uncharacterized, because appropriate culture conditions are lacking [63]. This Great Plate Count Anomaly is the oldest unresolved microbiological challenge. The Austrian microbiologist Heinrich Winterberg was the first who described this phenomenon in 1898 [64]. Winterberg observed that the number of microbial cells in his samples did not match the number of colonies formed on nutrient media. Since Winterberg, numerous authors who investigated bacterial communities in different habitats confirmed this phenomenon. The establishment of culture independent analytical methods in the early 1990s greatly expanded the dimension of knowledge about the bacterial diversity again [65]. Estimations, that about 80% of bacteria resist standard laboratory cultivation approaches were obsolete after publication of the first culture-independent analyses of bacterial communities, which were based on 16S rRNA-coding genes. Now, the estimated amount of uncultivable species has increased to 90–99% and it can be assumed that many of these uncultured bacteria could be probably a source for new antibiotics [66].

**Figure 4:**
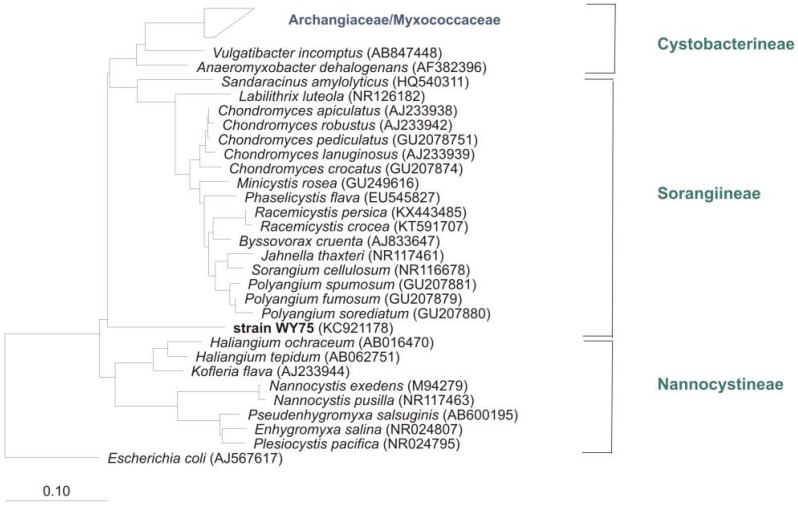
Neighbour joining tree with myxobacterial type strains shows the phylogenetic position of strain WY75, cultivated from ginger foundation soil, within the Sorangiineae suborder. Comparison of 16S rRNA sequences revealed only 87.4% similarity to the next myxobacterial type strain *S. amylolyticus*. Accession numbers are in brackets. Bar, 0.1 substitutions per nucleotide position.

Notwithstanding the frequent discovery and description of new species/genera, the real number of myxobacteria is unknown. The current knowledge about the diversity of organisms is always just a snapshot. However, several (NCBI) 16S rRNA-sequences of cultures belong to the order Myxococcales, but are only distantly related to valid type strains (up to 12% distance) and therefore probably belong to new species, genera, or even families. One example: “*Anaeromyxobacter dehalogenans*” strain WY75 (Acc. no. KC921178) was isolated from ginger foundation soil and shows highest similarity (87.4%) to the type strain of *Sandaracinus amylolyticus.* It is therefore at least a representative of a new myxobacterial family (Figure 4). Nevertheless, as long as a valid publication of such strains in taxonomic journals as for example *IJSEM* or *Antonie van Leeuwenhoek* is absent, even the current diversity of cultivable myxobacteria is not fully reflected.

**Figure 5:**
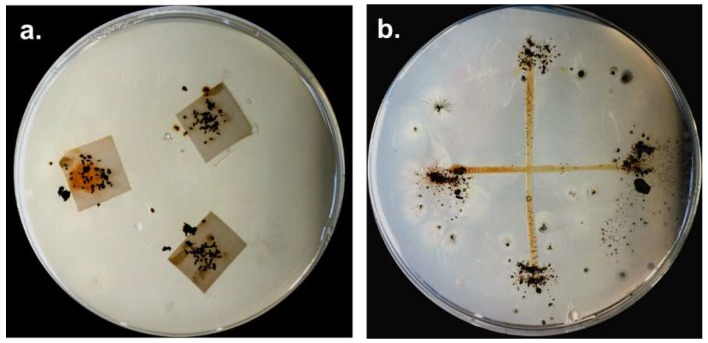
Common isolation procedure for myxobacteria: Soil/environmental sample is placed on **a.** Stan 21 with filter paper to enrich cellulose decomposing strains and **b.** on water agar with *E. coli* bait (cross) for predators. Numerous transfers of fruiting bodies/swarm edge material to fresh plates are necessary to purify myxobacteria.

Although there are numerous reports about cultivable myxobacteria in soils and other habitats [1], it has to be considered that myxospores may tolerate considerable environmental extremes. Most isolation techniques involve the cultivation of extensively dried samples [10]. Species which are present in the sample as vegetative cells will probably not survive this process and therefore will not grow on the isolation plates. Also, and irrespective of the detection method used, it is difficult to determine whether myxobacteria were present as dormant spores or metabolically active vegetative cells in the environmental sample taken [12]. The standard procedure to isolate myxobacteria is drying the sample (soil, plant material, etc.) at 30 °C to reduce growth of undesired bacteria and fungi, and subsequent placement on water agar with *E. coli*-bait (to attract predators) and on Stan 21 agar with filter paper (for cellulose decomposers), respectively (Figure 5). As the degradation of biomacromolecules like microbial cells (*E. coli* bait) or cellulose requires a sufficient amount of viable myxobacterial cells in the sample, underrepresented species will probably not be able to start growing.

As was mentioned at the beginning, myxobacteria live in various habitats. It is recommended, but not mandatory, to investigate uncommon habitats from different geographic regions with regard to new secondary metabolite producers. But, already within microscale areas of environmental samples, different strains of one myxobacterial species show surprising genetic differences, as biogeographical studies of myxobacteria revealed. Biogeography is the study of the distribution of organisms across space and time [67]. As mentioned by Ramette and Tiedje, prokaryotic biogeography is “the science that documents the spatial distribution of prokaryotic taxa in the environment at local, regional, and continental scales” [68]. Hanson et al. propose that four processes, selection, drift, dispersal, and mutation, create and maintain microbial biogeographic patterns on inseparable ecological and evolutionary scales [69]. For example, Bacteria and Archaea are globally distributed [70]. At the class level, the β-proteobacteria, cyanobacteria, actinobacteria, and flavobacteria have been shown to display worldwide distribution in marine or terrestrial ecosystems [71,72,73]. According to Hedlund and Staley, at the genus level, many prokaryotes have a cosmopolitan distribution in their respective habitats [74]. Recent global surveys indicate that most bacteria are restricted to broad habi­tat types, as there is little overlap among bacterial taxa found in soils, sediments, freshwater, and seawater [75,76]. Dawid gave a comprehensive overview about the ecology and global distribution of myxobacteria in the macroscale range [1]. The study was based on data given in the literature as well as on his own analyses of almost 1400 soil samples from 64 countries and all continents. The study found that an exceptionally high average species number was determined for soils from countries that belong to the winter rain climates of the Mediterranean type, the permanent wet rain forest climates and the tropical semi-desert climates. However, soils of countries with cold temperate coniferous forest climates and cool temperate intermediate climates with peat mosses and coniferous forests harbor a low average number of species. Jiang et al. determined biogeographic patterns of myxobacterial taxa in deep-sea sediments [77]. They screened DNA from four different depths for myxobacteria-like 16S rRNA genes and provided the first evidence, that marine myxobacteria are phylogenetically distinct from terrestrial species. Brinkhoff et al. studied the biogeography and phylogenetic diversity of marine myxobacteria and found a deep-branching monophyletic cluster of exclusively marine myxobacteria within the Myxococcales [78]. Wielgoss et al. sequenced the genomes of 22 *Myxococcus xanthus* isolates from a 16 × 16-cm-scale patch of soil. They found out “that two closely related *M. xanthus* clades inhabiting the same centimeter-scale patch of soil, display strong sexual isolation, with homologous recombination occurring frequently between members within each clade, but with almost no detectable levels of genetic exchange occurring across clades” [79]. Kraemer et al. resolved the micro biogeography of social identity and genetic relatedness in local populations of *M. xanthus* at small spatial scales [80]. The study comprises samples taken from fruiting bodies, neighboring fruiting bodies separated by millimeters, neighborhoods of fruiting bodies separated by centimeters and finally soil patches separated by meters and kilometers. They found out that “relatedness decreases greatly with spatial distance even across the smallest scale transition and that both, social relatedness and genetic relatedness are maximal within individual fruiting bodies at the micrometer scale but are much lower already across adjacent fruiting bodies at the millimeter scale.” What will this mean with regard to myxobacteria and natural product research? The cellulose degrading genus/species *Sorangium cellulosum* serves as an example: already in 2003, the myxobacterial strain collection of the HZI (former GBF) comprises 7000 strains from which 23.2% belong to *S. cellulosum.* On the other side, *S. cellulosum* strains produced 48.4% of all known secondary metabolites described so far from myxobacteria [81]. This means that closely related strains also have huge potential to produce different chemical and biological bioactive metabolites [48,49] and that the search for new antibiotic producers can be successful in both, small and large scale. For a comprehensive overview about biogeographic patterns of myxobacteria, I refer to Velicer et al. [82]. For a deeper insight to prokaryotic biogeography, see the study of Ramette and Tiedje [73] and the review of Hanson et al. [74].

With regard to numerous studies based on cultivation-dependent approaches, the number of publications that focus on the non-cultivable myxobacteria is comparatively small. Nevertheless, there are about 4000 (often unpublished) 16S rRNA sequences deposited at the NCBI database which are mentioned to be “uncultured Myxococcales”. Under consideration of further myxobacteria-related sequences which are just deposited as “uncultured (delta) proteobacterium” [83] or even “uncultured bacterium”, the true extent of uncultivated myxobacteria can just be surmised. Most of the deposited sequences are “by-products” from cultivation-independent studies of bacterial communities in general, without special focus on Myxococcales.

To give an impression about the diversity of cultivable and uncultivable myxobacteria in different habitats, published and unpublished 16S rRNA sequences from NCBI are compared with each other and the results are summarised subsequently.

## 5. Distribution of Myxobacteria in Different Habitats

### 5.1. Terrestrial Habitats

Myxobacteria are optimally adapted to terrestrial habitats, which manifests as a wide range of different phenotypes, such as social swarming and gliding, fruiting-body formation, resting myxospores, excretion of secondary metabolites with antibiotic or antifungal activity into the environment, as well as predation or cellulose decomposition. It is therefore not surprising that the majority of known species (and secondary metabolite producers) was isolated or detected from soil samples. In 1947, Singh investigated myxobacteria in soils and composts, their distribution, number, and lytic action on bacteria [44]. From soils of Great Britain, he isolated species of *Myxococcus*, *Chondrococcus* (later renamed to *Corallococcus*) and *Archangium* and estimated that the numbers of myxobacteria ranged from 2000 to 76,400/gram in soil. In an actively decomposing compost of sludge and straw, the number of *Myxococcus fulvus* was more than 500,000/g. Singh was also the first who detected the potential of myxobacteria to produce antibiotics.

In 2005, Wu et al. were the first to explore the diversity of myxobacteria (in a soil niche) by cultivation-independent methods with myxobacteria-specific primers and probes [84]. Moreover, in the latter study members of *Myxococcus*, *Corallococcus*, *Cystobacter*, and *Nannocystis* were cultivated. Nevertheless, screening a special library using Cystobacterineae- and Sorangiineae-specific probes and subsequent sequence analyses revealed a somewhat higher number of myxobacteria within the sample, from which many show only minor similarity to known species. Therefore, even in this first cultivation-independent study about Myxococcales, the authors suggested that myxobacteria in nature are much more diverse than were ever known, even in a single soil sample.

Jiang et al. investigated fruiting and non-fruiting myxobacteria and gave a phylogenetic perspective of cultured and uncultured members of this group [85]. The authors analysed the diversity of myxobacteria in campus garden soil and found out that many undescribed relatives exist in nature and concluded that there are two forms: the fruiting and the non-fruiting types. They postulated that most of the uncultured myxobacteria might represent taxa, which rarely form fruiting bodies, or may lack some or all of the developmental genes needed for fruiting body formation. The majority of sequences from the cultivation-independent approach are only distantly related to known genera and species. As myxobacteria are widespread in terrestrial habitats, consequently, they are frequently detected in those cultivation-independent studies on microbial diversity. Even in uncommon habitats like adult worker ants myxobacteria were detected [86].

**Figure 6:**
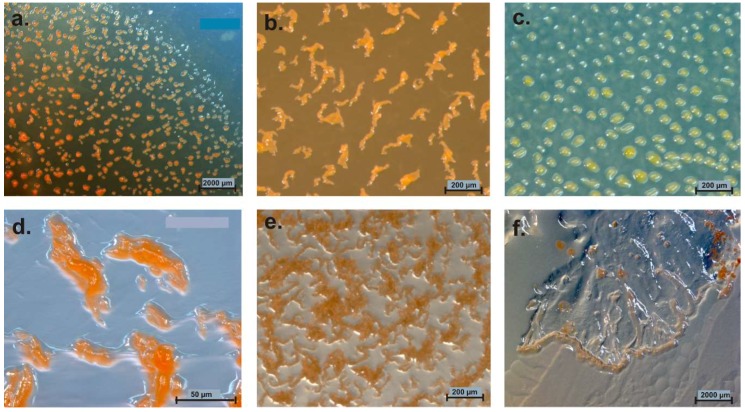
Myxobacterial cultures isolated from Kiritimati sand (**a**,**d**) and German compost (**b**–**f**) modified from Mohr et al. [9]. (**a**) *Corallococcus* (*Myxococcus*) *macrosporus*, (**b**) *Corallococcus* sp., (**c**) *Myxococcus* sp. (**d**) *Archangium gephyra*, (**e**) *Corallococcus* sp., (**f**) *Polyangium fumosum.*

In our study about myxobacteria in two geographically distant locations, namely sand from Kiritimati Island and German compost, we also compared the diversity of cultivable myxobacteria to those from cultivation-independent clone libraries [9]. The study revealed an overrepresentation of the genera *Myxococcus* and *Corallococcus* with standard cultivation methods (Figure 6).

However, phylogenetic analyses of the 16S rRNA gene sequences from clones revealed a great potential of undescribed myxobacteria in both sampling sites. Several OTUs (operational taxonomic units; groups of sequences with ≥97% similarity) represented unknown taxa exclusively detected by cultivation-independent analyses, but not by cultivation. Furthermore, clone library analyses indicated that the myxobacterial community of the investigated samples is predominantly indigenous.

Most of the known myxobacterial secondary metabolites were previously isolated from terrestrial myxobacteria, because the majority of strains was isolated from (moderate) terrestrial habitats. However, myxobacteria are extremely adaptable and can also be found in demanding environments like acidic soils, fresh water, oceans and salines, anaerobic/microaerophilic, and extreme habitats, respectively.

### 5.2. Acidic and Alkaline Habitats

Acidic wetlands have a major impact on the global carbon and water cycles. With high acidity (pH 3.5 to 5.0), low temperatures, and extremely low concentrations of mineral nutrients (5 to 50 mg per liter), wetlands are moderate to extreme habitats. Their microbial diversity remains poorly understood, because only microbial populations involved in CH_4_ cycling, i.e., methanotrophic bacteria and methanogenic archaea, have attracted considerable research interest. Other members of the microbial communities in acidic *Sphagnum* peatlands remain largely unknown [87].

The pH range for growth of the majority of myxobacteria is rather narrow, approximately 6.5–8.5. Therefore, they are common in soils of pH 6–8 (neutral to slightly alkaline pH). However, acidic or alkaline habitats also seem to be suitable for myxobacteria [10]. Even in 1977, Hook isolated ten species from waters of an alkaline bog and adjacent soils [88] like *Archangium*, *Corallococcus*, *Melittangium, Myxococcus*, and *Sorangium* (former *Polyangium*). *Corallococcus coralloides*, (formerly *Myxococcus coralloides*) was dominant in the terrestrial samples. With pH between 6.0 and 8.7, the investigated habitats were between slightly acidic and slightly alkaline. In 1979, Rückert also described *C. coralloides* as the predominant species in soils of pH 4.1–4.9 and as dominant as *M. fulvus* in soils of pH 3.0–3.5 [89]. In alpine acidic soils *C. coralloides* was the third-most dominant species behind two *Myxococcus* species. But, Rückert also noted that the overall myxobacterial diversity in acidic soils (pH 3.5–4.9) was less than in slightly acidic or neutral environments (pH 5.0–7.8). In 1984, Dawid isolated *Myxococcus xanthus, M. virescens* and *Polyangium* sp., but no cellulolytic species from undisturbed *Sphagnum* bogs of the Hohen Venn, Belgium [3].

**Figure 7:**
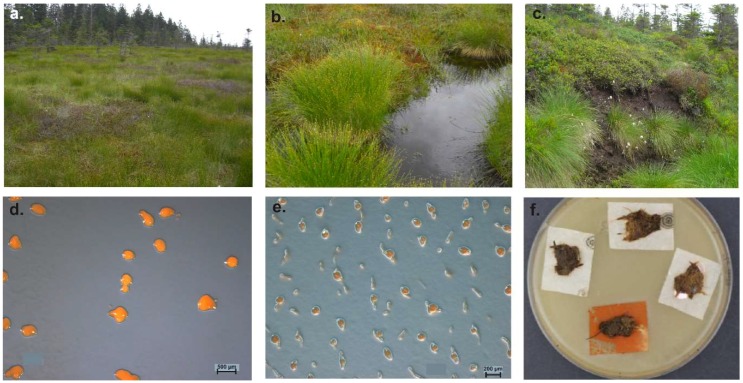
(**a**) Brockenfeld high moor; (**b**) fen Am Sandbeek; (**c**) Brockenfeld high moor scarp. Isolated *Corallococcus* sp. strains from moors (**d**) strain B19, (**e**) strain B2t-1. (**f**) *Sorangium cellulosum* (orange) on a raw culture plate (Stan 21 with filter) inoculated with soil material from moor. Pictures are from Mohr et al. (2017) and modified [2].

Mohr et al. studied myxobacteria in peat bog and fen with cultivation and cultivation-independent methods [2]. Therefore, 38 moor samples of soil, water, plant residues, mud, and feces-material (Figure 7a–c) were screened using standard as well as moor-adjusted cultivation conditions (low pH, low temperature, moor-water for preparation of agar plates), screening numerous replicates over several years. The pH of moor samples analyzed in this study was between 4.0 and 7.0 and therefore comparable to those from the other studies. But almost exclusively species of the genus *Corallococcus* could be isolated from acidic soils of the Harz-region (Figure 7d,e). A *Sorangium* strain was detected on a raw culture plate with filter (Figure 7f), but could not be purified. In addition, the community composition of acidic high moor and fen revealed by cultivation-independent 16S rRNA clone library analysis gave a rather different picture of the myxobacterial diversity.

**Figure 8:**
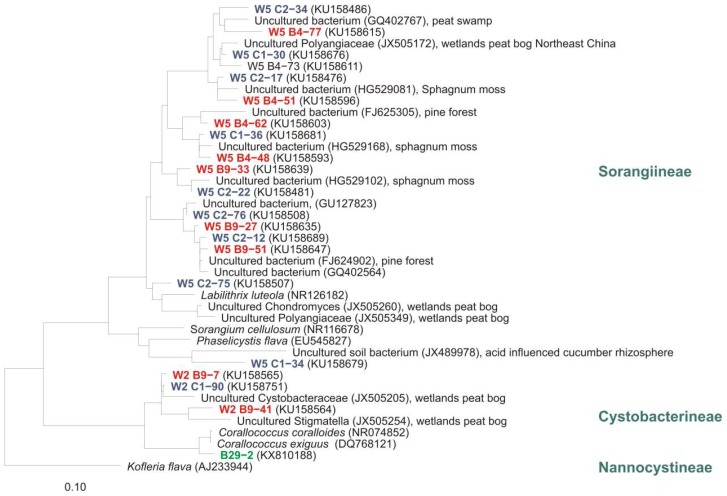
Part of a distance tree showing some myxobacterial type strains, some representative clone sequences and one representative culture sequence from our study [2] as well as sequences from uncultivated myxobacteria from other studies. Red: clones from Brockenfeld high moor; blue: clones from fen Am Sandbeek; green: representative culture from Brockenfeld high moor. Accession numbers are given in brackets. Origin of sequences from uncultured myxobacteria are mentioned. Bar, 0.1 substitutions per nucleotide position.

Phylogenetic analyses of clone sequences revealed a high diversity of undescribed myxobacteria in high moor and fen. Many sequences represent totally unknown taxa. However, numerous clones were closely related to sequences from other cultivation-independent studies of eubacterial diversity in which samples from peat swamp, wetlands peat bog, *Sphagnum* moss, pine forest, acidic fen soil, and forest soil were analysed (Figure 8). As mentioned above, cultivation exclusively revealed strains from the genus *Corallococcus* (but from almost all analysed samples).

To my knowledge, no publications about (bioactive) secondary metabolites from myxobacteria isolated from acidic or alkaline habitats are available. However, we screened 21 *Corallococcus* spp.-strains from the moor study for production of bioactive metabolites. Raw extracts of all strains showed high activity against Gram positives (*Micrococcus luteus*, *Staphylococcus aureus*, *Bacillus subtilis*, and *Mycobacterium* sp.), the yeasts *Saccharomyces pombe* and *Rhodotorula glutinis*, as well as against the filamentous fungi *Mucor hiemalis*, but no activity against Gram negatives. HPLC analyses of the raw extracts revealed three dominant peaks. By HPLC-fractionation of bioactive extracts and subsequent HPLC-MS analyses the already known substances dibenzylpyrazine, myxothiazol A, and myxothiazol Z/A-methylester, respectively, were identified (data not published). In summary, the moor habitat is a promising source and of high interest with regard to the cultivation of prospective new bioactive secondary metabolite-producing myxobacteria.

### 5.3. Freshwater Habitats

In natural aquatic environments, microbial cells often build complex, surface-attached biofilm communities. Within the water body or pelagic zone of unpolluted freshwaters, the number and diversity of bacteria is normally lower than on the available substrates. Myxobacteria glide in swarms over solid surfaces. If it is possible, they prefer attached in contrast to planktonic living.

Only very few studies about myxobacteria in fresh water habitats are published. In the 1960th/1970th several studies dealt with nonpathogenic or pathogenic non-fruiting “myxobacteria” as colonizers of freshwater fish [90]. However, these publications deal with strains of the Cytophaga-group, which do not belong to the Myxococcales, but to the Cytophaga—Flavobacterium—Bacteroides group. No myxobacterial pathogens are published. Reichenbach mentioned that myxobacteria can also be isolated from fresh water, but explained these findings with soil organisms notoriously exchange into water bodies, being regularly washed or blown in and often surviving there periodically or permanently [12].

In 2012, Li and co-workers investigated the myxobacterial community in freshwater lake mud using high-throughput 454 pyrosequencing and myxobacteria-enriched libraries with Cystobacterineae- and Sorangiineae-specific primer pairs, respectively, and reported that myxobacteria were one of the major bacterial groups in the lake mud [91]. Phylogenetic analysis showed that the limnetic myxobacteria exhibit closer relationships to their soil than to their marine relatives, but there are also exclusive taxa of limnetic myxobacteria. The major conclusion was that the unclassified Myxococcales in the lake mud comprise a large portion of the microbiota and exhibit high species diversity. Kou et al. analysed bacterial communities in sediments of freshwater (Poyang Lake) in China. There, *Anaeromyxobacter dehalogenans* turned out to be a main part of the bacterial community composition (1–14.6%) [92]. In another study about methanogenic microbial communities in sediments of Amazonian lakes using terminal restriction fragment length polymorphism (T-RFLP) and pyrosequencing, the proteobacteria revealed as the most abundant phylum in all lake sediments. Delta-proteobacteria (mainly Myxococcales, Syntrophobacteriales and sulfate/sulfur-reducing bacteria) dominated this habitat [93]. In 2014, Kandel et al. investigated the abundance, diversity, and seasonal dynamics of predatory bacteria in aquaculture zero discharge systems by cultivation-independent analyses and found out that in addition to the detected *Bdellovibrio* and similar organisms, other potential predators were highly abundant, especially from the Myxococcales [94].

In the absence of cultures which are verifiable natural fresh water inhabitants, up to now, no (bioactive) metabolites have been isolated from limnic strains. However, the above-mentioned detection of exclusively limnic taxa [95] suggest that also the habitat fresh water could be a promising source for the cultivation of new secondary metabolite producing myxobacteria.

### 5.4. Marine/Saline Environments

**Figure 9:**
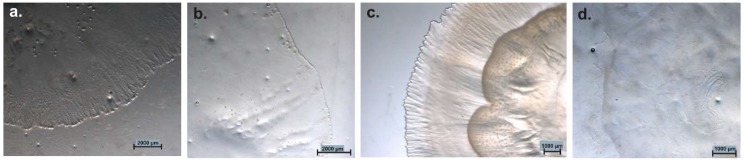
Marine myxobacterial type strains on agar plates. (**a**) *Haliangium tepidum* (DSM 14436T) on VY/2SWS, (**b**) *Enhygromyxa salina* (DSM 15217T) on VY/4SWS, (**c**) *Pseudenhygromyxa salsuginis* (DSM 21377T) on 1102, (**d**) *Plesiocystis pacifica* (DSM 14875T) on VY/2SWS.

Covering more than roughly 78% of the earth’s surface, water is the most prevalent natural substance, of which approximately 97.5% is salt water in the world’s oceans [96]. The salt tolerance of myxobacteria is low in general. It was assumed for a long time that myxobacteria exclusively live in terrestrial habitats. Indeed, even in 1963, Brockman observed fruiting myxobacteria in sand samples from an ocean beach in South Carolina [97]. Species of the already known terrestrial genera *Archangium*, *Chondrococcus* (*Corallococcus*), *Chondromyces*, *Myxococcus*, and *Polyangium*, could be cultivated. As late as 2002 with *Haliangium ochraceum* and *H. tepidum*, the first myxobacterial genus was isolated and described from coastal salt marshes. The strains differ from known terrestrial myxobacteria with regard to salt requirements (2–3% NaCl) and the presence of anteiso-branched fatty acids [4]. Other genera, exclusively detected in marine habitats like *Plesiocystis* [5], *Enhygromyxa* [6], and *Pseudenhygromyxa* [7] (all Nannocystineae-suborder) followed (Figure 9).

In 2010, Jiang et al. investigated the diversity of marine myxobacteria in comparison to terrestrial soil myxobacteria [82]. Therefore, they established myxobacteria enriched libraries of 16S rRNA gene sequences from four deep-sea sediments and a hydrothermal vent and identified 68 different myxobacteria related sequences from randomly sequenced clones of these libraries. The authors concluded that the myxobacterial sequences were diverse but phylogenetically similar at different locations and depths. However, they separate from terrestrial myxobacteria at high levels of classification.

**Figure 10:**
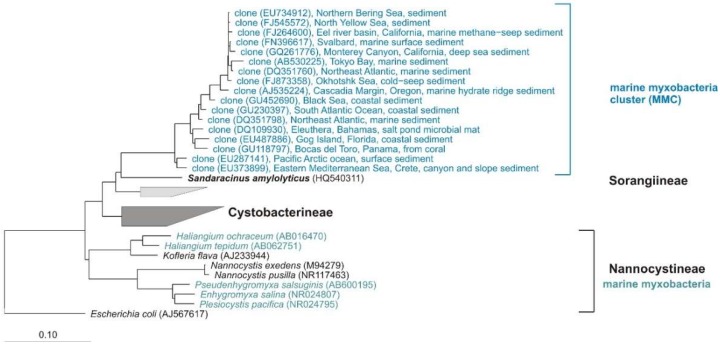
Neighbour joining tree of myxobacterial type strains (16S rRNA-genes), some representative clones from the MMC-cluster (blue) and the next cultivated relative *Sandaracinus amylolyticus*. Genera isolated from marine environment are in green blue. Suborders of the order Myxococcales, origin of clones, and accession numbers are shown. Bar, 0.1 substitutions per nucleotide position.

In 2012, the study of Brinkhoff and co-workers gave an impressive insight to the marine myxobacterial community [83]. They detected a cluster of exclusively marine myxobacteria (marine myxobacteria cluster, MMC) in sediments of the North Sea, but not in the limnetic section of the Weser estuary and other freshwater habitats. In a quantitative real-time PCR approach, the authors found out that the MMC constituted up to 13% of total bacterial 16S rRNA genes in surface sediments of the North Sea. In addition, in a global survey including sediments from the Mediterranean Sea, the Atlantic, Pacific, and Indian Oceans, and various climatic regions, the MMC appears in most samples and to a water depth of 4300 m, but there was no synteny to other myxobacterial genomes. The study of Brinkhoff et al. showed that the MMC is an important and widely distributed but largely unknown component of marine sediment-associated bacterial communities. Figure 10 shows some representative clones from different marine habitats mentioned in the Brinkhoff study. The 17 clones show 95.1–100% similarity to each other and 88–91% to the next type strain *Sandaracinus amylolyticus.* This implies that members of the MMC cluster do at least belong to new genera if not even families, but presumably belong to the Sorangiineae suborder. However, the genus *Sandaracinus* was published shortly after the Brinkhoff study, so this relative was not mentioned there.

In 2013, Zhang et al. isolated fifty-eight terrestrial and salt-tolerant myxobacteria from the saline-alkaline soils collected from Xinjiang, China [95]. Based on morphology and 16S rRNA gene sequences, the authors identified species of *Myxococcus*, *Cystobacter*, *Corallococcus*, *Sorangium*, *Nannocystis*, and *Polyangium*. They reported that all the strains grew better with 1% NaCl than without salt; some *Myxococcus* strains even grow with 2% NaCl.

Li et al. (2014) chose a cultivation-independent approach to analyze the diversity of myxobacteria from saline-alkaline soils of Xinjiang, China, too. A semi-nested PCR-denaturing gradient gel electrophoresis (DGGE) based on the taxon-specific gene mglA (a key gene involved in gliding motility) was used [98]. In accordance to previous studies, Li et al. also suggested that there are still many viable, but under standard laboratory conditions uncultured myxobacterial strains in the investigated saline-alkaline habitat. Natural product classes discovered from marine Myxococcales strains include polyketides, hybrid polyketide-nonribosomal peptides, degraded sterols, diterpenes, cyclic depsipeptides, and alkylidenebutenolides [99]. Four genera of marine/saline origin are known so far (Figure 10) and from two, *Haliangium* and *Enhygromyxa*, numerous (bioactive) secondary metabolites could be isolated [100]: Haliangicin [101], salimabromide [102], salimyxins, enhygrolides [103], and haliamide [104]. The above-mentioned data reveal that marine/saline environments as oceans harbor an enormous potential of new myxobacteria. These organisms are an unexplored resource of novel antibiotics of novel chemical scaffolds, as mentioned by Albataineh and Stevens, who highlighted the need for continued discovery and exploration of marine myxobacteria as producers of novel natural products [104].

### 5.5. Facultative or Strictly Anaerobic Myxobacteria

All known myxobacteria live aerobically, with one exception: The facultative-anaerobic genus *Anaeromyxobacter* comprises one species, *A. dehalogenans.* The type strain was isolated from stream sediment and grows with acetate as electron donor and 2-chlorophenol (2-CPh) as electron acceptor [11]. Since 2002, several strains of *Anaeromyxobacter* were isolated from various habitats. Flooded rice field soil [105], uranium contaminated surface environment [106], corrosion material of drinking water pipelines [107], arsenic-contaminated soils [108], or chemically and electro-chemically enriched sodic-saline soil (unpublished) served as sources for the cultivation of *Anaeromyxobacter*-strains. A total of 23 sequences designated as *Anaeromyxobacter* (sequence lengths > 1000 bp) are available from the NCBI database (FJ90053–FJ90062, FJ90048, FJ90049, FJ90051, EF067314, AJ504438, KF952446, AF382397, AF382399, AF382400, FJ939131, KF952441, KF952438, KC921178) and were added to a phylogenetic tree of myxobacterial type strains. A similarity matrix calculated with arb (www.arb-home.de) revealed 98.4–100% similarity (on basis of 16S rRNA gene) for 20 of these strains to the type strain of *A. dehalogenans*. Assuming that the standard value for the definition of a new species is 98.65% [109] and 94% for a new genus, respectively, the above-mentioned 20 cultures probably do belong to *A. dehalogenans*. However, strains OnlyC-B2 (KF952441) and SSS-B8 (KF952438) show only 96.3% and 96.0% similarity to the corresponding type strain (but 99.4% to each other) and putatively represent a new species. One culture, isolated from ginger foundation soil, and also designated as *A. dehalogenans* (KC921178), shows only 86% similarity to the next cultivated (myxobacterial) type strain *Vulgatibacter incomptus*. This culture definitely represents at least a new family if not even a new suborder of myxobacteria (unpublished).

In summary, there are currently two cultivated species of *Anaeromyxobacter,* but only one is validly described. But what about further facultative or even strictly anaerobic myxobacteria?

The NCBI search for 16S rRNA gene sequences of “uncultured *Anaeromyxobacter*” revealed more than 1200 hits. Nevertheless, not all sequences designated as “Uncultured *Anaeromyxobacter*” are close relatives of *Anaeromyxobacter*, as a revision of randomly selected sequences revealed. For example: the sequence GU271851, mentioned as “Uncultured *Anaeromyxobacter*”, shows 91% similarity to the next type strain *Haliangium tepidum,* but only 87% to the type strain of *A. dehalogenans* [110]. Clone GU271788, also mentioned as *A. dehalogenans*, shows 92% to the next type strain, *Sorangium cellulosum*, but only 86% to *Anaeromyxobacter* [111]. On the other hand, there are probably numerous sequences deposited at NCBI which are close relatives of *Anaeromyxobacter*. However, these sequences are just mentioned as “uncultivated Myxococcales”, “uncultivated (delta) proteobacteria” or “uncultured bacterium clone” such as clone EUB_19 (FJ189540), which shows 98.7% [112] or clone A_Ac-2_16 (EU307085), which shows 97.8% similarity to the next type strain: *A. dehalogenans* [113]. In 2009, Thomas et al. analysed the diversity and distribution of *Anaeromyxobacter* strains in a uranium-contaminated environment by mainly cultivation-independent methods. Phylogenetic analyses of the clone and culture sequences revealed that there are at least three distinct *Anaeromyxobacter* clusters at the IFC (Integrated Field-Scale Subsurface Research Challenge) site near Oak Ridge, whereby two sides are exclusively represented by clones. As mentioned above, quantitative PCR assay and pyrosequencing analysis of 16S rRNA genes also revealed *A. dehalogenans* as a part of the microbial community in the sediment of Poyang Lake, the largest freshwater lake in China [95].

**Figure 11:**
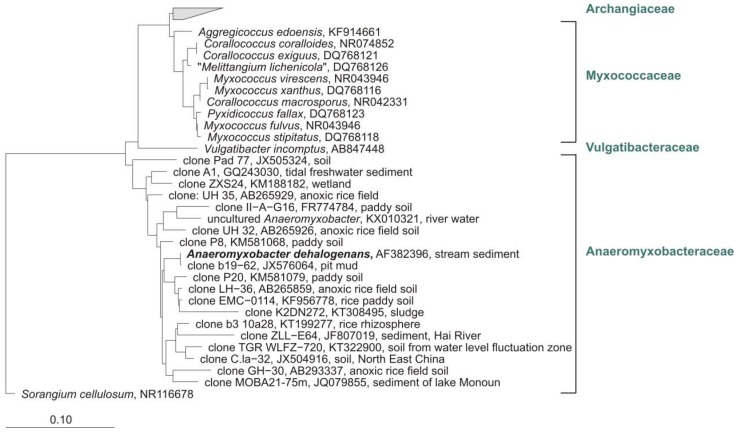
Neighbour joining tree of some myxobacterial type strains (16S rRNA-genes) and some representative clones. All clones show highest relationship to the next cultivated relative *A. dehalogenans*. All families of the Cystobacterineae suborder, origin of samples, and accession numbers of clones/cultures are shown. Bar, 0.1 substitutions per nucleotide position.

To get an impression about the diversity of uncultured *Anaeromyxobacter*, I added about 80 clone sequences from NCBI with corresponding designation to a phylogenetic tree of myxobacterial type strains (data not shown). The clones revealed 90.0–100% similarity on the basis of 16S rRNA gene to the type strain of *A. dehalogenans* (AF382396). Figure 11 shows the affiliation of some representative clones. These clones represent at least several new genera, if not even families of myxobacteria, which are probably also facultative or strictly anaerobic and which could not be cultivated so far.

Although the 16S affiliation of clones does not give any information about metabolism of the corresponding organism, high similarities to aerobic or anaerobic cultures indicate similar metabolic capabilities. However, no bioactive secondary metabolites have been described so far from *Anaeromyxobacter* strains, which is certainly because anaerobic isolation, cultivation, and large scale fermentation requires special efforts regarding equipment and microbiological skills and experience.

### 5.6. Moderate to Extreme Hot or Cold Environments

**Figure 12:**
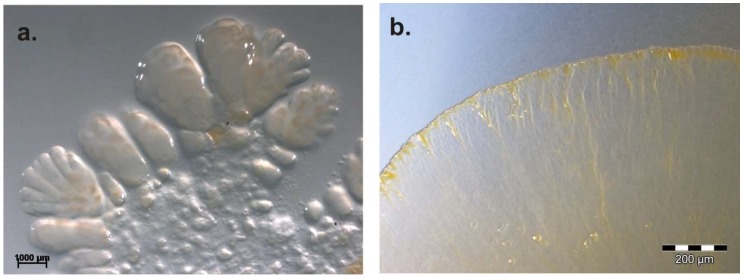
Moderately thermophilic strains of *Sorangium* on VY/2 agar. (**a**) GT47 and (**b**) GT 41, isolated by Dr. K. Gerth.

Myxobacteria are mesophilic and grow well at 30 °C, although their temperature range is much wider. For most myxobacterial strains, the growth temperatures is between 4 °C and 44 °C. Usually vegetative cells cannot survive temperatures above 45 °C, but myxospores suspended in water tolerate 58–60 °C. This property can be used to be purify myxobacteria from mesophilic accompaniment organisms [12]. A moderate terrestrial habitat was investigated by Brockman in 1976 [114], who isolated strains of *Archangium*, *Chondromyces, Cystobacter*, *Myxococcus*, *Polyangium,* and *Stigmatella* from arid Mexican soils. He reported a greater species diversity from regions with higher annual rainfall (400–800 mm compared to 200–400 mm). Moderate thermophilic myxobacteria of Cystobacterineae and Sorangiineae-suborders, which grew very fast at temperatures of 42 °C–44 °C, were isolated from soil samples of semiarid and warm climates by Gerth and Müller [115] (Figure 12). One strain even grew at 48 °C, whereas the majority of the described species grows best at 30 °C.

**Figure 13:**
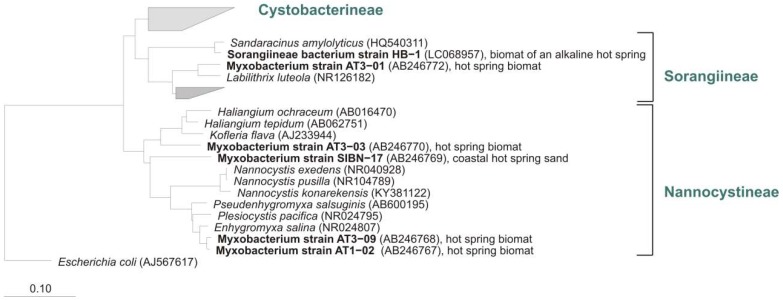
Neighbour joining tree of some myxobacterial type strains (16S rRNA-genes) and cultures (in bold) from hot springs (AB246767-AB246770, AB246772) [117] and alkaline hot spring, all from Japan. Suborders of the order Myxococcales, origin of samples, and accession numbers are shown. Bar, 0.1 substitutions per nucleotide position.

Recently, a new *Nannocystis* species, *N. konarekensis*, was isolated from an Iranian desert [111]. The strain shows an optimal growth temperature at 37 °C, in contrast to the other known *Nannocystis* species *N. pusilla* and *N. exedens*, which show optimal growth at 30 °C. Iizuka et al. reported about enrichment and phylogenetic analysis of moderately thermophilic myxobacteria. During their search for thermophilic myxobacteria in geothermal environments, four strains that grew at temperatures up to 50 °C (optimum 45 °C–49 °C) could be isolated from various hot springs in Japan [116]. Three of the cultures were from fresh water hot springs and one was from a coastal saline spring. Even after repeated enrichment procedures, other thin film-like spreading bacteria accompanied the strains. PCR, cloning, and sequencing of 16S genes revealed that all cultivated bacteria belong to the order Myxococcales and showed between 89–99% homology to strains of myxobacteria. Therefore, some of these cultures represent new undescribed but cultivable species, genera, and perhaps even families (Figure 13).

Although numerous (cultivation-independent) studies about bacterial diversity of hot springs/geothermal sources are published, the NCBI search for sequences of uncultured thermophilic myxobacteria or myxobacteria from hot springs revealed only very few matches. Hot springs are probably not the most suitable habitat for the mainly mesophilic myxobacteria. But, based on the cultivation success mentioned by Iizuka et al. [116], it is certainly worth investigating these habitats more precisely to isolate new myxobacteria.

Some publications deal with myxobacteria from cold environments like Arctic soils. However, in the study of Brockman who tried to isolate myxobacteria from Alaskan and Canadian Arctic soils, myxobacterial growth was only observed when soil plates were incubated at 24 °C–26 °C, but not at 6 °C–8 °C [118]. In contrast, Dawid described psychrophilic myxobacteria which grow at 4 °C but not under mesophilic conditions between 18 °C and 30 °C (after 7–9 month of incubation) on samples of Antarctic soils [117].

Due to long incubation times of psychrophilic strains, their biotechnological use in large scale fermentation would be expensive and time consuming and would only be worthwhile if a highly promising antibiotic was detected in such a psychrophilic strain.

## 6. Conclusion

In summary, myxobacteria are highly adaptable cosmopolitans. They can grow/survive in various kind of habitats and areas of different, even extreme climatic conditions. In 1993, only 2 suborders, 4 families, 12 genera, and 38 species were assigned to the order Myxococcales [28], but in 2018, already 3 suborders, 10 families, 29 genera, and 58 species are described. Although the number of species grows every year, consideration of data from cultivation-independent studies reveals that we only see the tip of the diversity iceberg.

In 2010, 67 distinct core structures and about 500 derivatives were known from approximately 7500 myxobacterial strains [48]. Only seven years later, Herrmann et al. could refer to five natural product classes produced by myxobacteria [49]. These new molecules show such promising activity that several of them may serve as early lead structures for drug development. This shows the enormous potential of myxobacteria as producers of new, bioactive secondary metabolites. As mentioned by Müller and Wink, three of the most promising approaches toward finding novel anti-infectives from microorganisms are the use of biodiversity to find novel producers, the variation of culture conditions and induction of silent genes, and the exploitation of the genomic potential of producers via “genome mining” [119]. With focus on novel producers, the biggest challenge for microbiologists is to get access to the so far uncultivated bacteria.

## References

[1] Dawid W. (2000). Biology and global distribution of myxobacteria in soils. FEMS Microbiol. Rev..

[2] Mohr K.I., Zindler T., Wink J., Wilharm E., Stadler M. (2017). Myxobacteria in high moor and fen: An astonishing diversity in a neglected extreme habitat. MicrobiologyOpen.

[3] Dawid W. (1984). Myxobakterien in ungestörten Hochmooren des Hohen Venn (Hautes Fagnes, Belgien). Syst. Appl. Microbiol..

[4] Fudou R., Jojima Y., Iizuka T., Yamanaka S. (2002). *Haliangium ochraceum* gen. nov., sp. nov. and *Haliangium tepidum* sp. nov.: Novel moderately halophilic myxobacteria isolated from coastal saline environments. J. Gen. Appl. Microbiol..

[5] Iizuka T., Jojima Y., Fudou R., Hiraishi A., Ahn J.W., Yamanaka S. (2003). *Plesiocystis pacifica* gen. nov., sp. nov., a marine myxobacterium that contains dihydro-genated menaquinone, isolated from the Pacific coasts of Japan. IJSEM.

[6] Iizuka T., Jojima Y., Fudou R., Tokura M., Hiraishi A., Yamanaka S. (2003). *Enhygromyxa salina* gen. nov., sp. nov., a slightly halophilic myxobacterium isolated from the coastal areas of Japan. Syst. Appl. Microbiol..

[7] Iizuka T., Jojima Y., Hayakawa A., Fujii T., Yamanaka S., Fudou R. (2013). *Pseudenhygromyxa salsuginis* gen. nov., sp. nov., a myxobacterium isolated from an estuarine marsh. IJSEM.

[8] Menne B., Rückert G. (1988). Myxobakterien (Myxobacterales) in Höhlensedimenten des Hagengebirges (Nördliche Kalkalpen). Die Höhle. Z Karst Höhlenkd.

[9] Mohr K.I., Stechling M., Wink J., Wilharm E., Stadler M. (2016). Comparison of Myxobacterial Diversity and Evaluation of Isolation Success in two niches: Kiritimati Island and German Compost. MicrobiologyOpen.

[10] Shimkets L.J., Dworkin M., Reichenbach H., Dworkin M., Falkow S., Rosenberg E., Schleifer K.H., Stackebrandt E. (2006). The Myxobacteria. The Prokaryotes.

[11] Sanford R.A., Cole J.R., Tiedje J.M. (2002). Characterization and Description of *Anaeromyxobacter dehalogenans* gen. nov., sp. nov., an Aryl-Halorespiring Facultative Anaerobic Myxobacterium. AEM.

[12] Reichenbach H. (1999). The ecology of the myxobacteria. Environ. Microbiol..

[13] Nan B., Chen J., Neu J.C., Berry R.M., Oster G., Zusman D.R. (2011). Myxobacteria gliding motility requires cytoskeleton rotation powered by proton motive force. Proc. Natl. Acad. Sci. USA.

[14] Kaiser D., Robinson M., Kroos L. (2010). Myxobacteria, Polarity, and Multicellular Morphogenesis. Cold Spring Harb. Perspect. Biol..

[15] Mauriello E.M.F., Mignot T., Yang Z., Zusman D.R. (2010). Gliding Motility Revisited: How Do the Myxobacteria move without Flagella?. Microbiol. Mol. Biol. Rev..

[16] Faure L.M., Fiche J.B., Espinosa L., Ducret A., Anantharaman V., Luciano J., Lhospice S., Islam S.T., Tréguier J., Sotes M. (2016). The mechanism of force transmission at bacterial focal adhesion complexes. Nature.

[17] Burchard R.P., Dworkin M. (1966). Light-induced lysis and carotenogenesis in *Myxococcus xanthus*. J. Bacteriol..

[18] Zusman D.R., Scott A.E., Yang Z., Kirby J.R. (2007). Chemosensory pathways, motility and development in *Myxococcus xanthus*. Nat. Rev. Microbiol..

[19] Thaxter R. (1892). On the Myxobacteriaceae, a new order of the Schizomycetes. Bot. Gaz..

[20] Kofler L. (1913). Die Myxobakterien der Umgebung von Wien. Sitzungsberichte der Akademie der Wissenschaften mathematisch-naturwissenschaftliche Klasse.

[21] Baur E. (1904). Myxobakterienstudien. Arch. Protistenkunde.

[22] Jahn E. (1924). Beiträge zur botanischen Protistologie.

[23] Jahn E. (1936). Kulturmethoden und Stoffwechseluntersuchungen bei Myxobakterien (Polyangiden).

[24] Kühlwein H. (1950). Beiträge zur Biologie und Entwicklungsgeschichte der Myxobakterien. Arch. Mikrobiol..

[25] Reichenbach H., Höfle G. (1993). Biologically active secondary metabolites from myxobacteria. Biotechnol. Adv..

[26] Mohr K.I., Stadler M., Dersch P. (2017). History of antibiotics research. How to Overcome the Antibiotic Crisis—Facts, Challenges, Technologies & Future Perspective.

[27] Houbraken J., Frisvad J.C., Samson R.A. (2011). Fleming’s penicillin producing strain is not *Penicillium chrysogenum* but *P. rubens*. IMA Fungus.

[28] Karwehl S., Stadler M., Stadler M., Dersch P. (2017). Exploitation of fungal biodiversity for discovery of novel antibiotics. How to Overcome the Antibiotic Crisis—Facts, Challenges, Technologies & Future Perspective.

[29] Waksman S.A., Woodruff H.B. (1940). Bacteriostatic and bacteriocidal substances produced by soil actinomycetes. Proc. Soc. Exp. Biol..

[30] Schatz A., Bugie E., Waksman S. (1944). Streptomycin: A substance exhibiting antibiotic activity against gram positive and gram negative bacteria. Proc. Exp. Biol. Med..

[31] Duggar B.M. (1948). Aureomycin: A product of the continuing search for new antibiotics. Ann. N. Y. Acad. Sci..

[32] McGuire J.M., Bunch R.L., Anderson R.C., Boaz H.E., Flynn E.H., Powell H.M., Smith J.W. (1952). Ilotycin, a new antibiotic. Antibiot. Chemother..

[33] Drews J. (2000). Drug discovery: A historical perspective. Science.

[34] Aminov R.I. (2009). The role of antibiotics and antibiotic resistance in nature. Environ. Microbiol..

[35] Bartlett J.G., Gilbert D.N., Spellberg B. (2013). Seven ways to preserve the miracle of antibiotics. Clin. Infect. Dis..

[36] Ventola C.L. (2015). The Antibiotic Resistance Crisis: Part 1: Causes and Threats. Pharm. Ther..

[37] Hesterkamp T., Stadler M., Dersch P. (2016). Antibiotics Clinical Development and Pipeline. How to Overcome the Antibiotic Crisis—Facts, Challenges, Technologies & Future Perspective.

[38] Hoffmann T., Krug D., Bozkurt N., Duddela S., Jansen R., Garcia R., Gerth K., Steinmetz H., Müller R. (2018). Correlating chemical diversity with taxonomic distance for discovery of natural products in myxobacteria. Nat. Commun..

[39] Landwehr W., Wolf C., Wink J., Stadler M., Dersch P. (2016). Actinobacteria and Myxobacteria—Two of the Most Important Bacterial Resources for Novel Antibiotics. How to Overcome the Antibiotic Crisis—Facts, Challenges, Technologies & Future Perspective.

[40] Sansinenea E., Ortiz A. (2011). Secondary metabolites of soil *Bacillus* spp.. Biotechnol. Lett..

[41] Singh B.N. (1947). Myxobacteria in Soils and Composts; their Distribution, Number and Lytic Action on Bacteria. Microbiology.

[42] Mathews S., Dudani A. (1955). Lysis of human pathogenic bacteria by myxobacteria. Nature.

[43] Noren B., Raper K.B. (1962). Antibiotic activity of myxobacteria in relation to their bacteriolytic capacity. J. Bacteriol..

[44] Ringel S.M., Greenough R.C., Roemer S., Connor D., Gutt A.L., Blair B., Kanter G., von Strandtmann M. (1977). Ambruticin (W7783), a new antifungal antibiotic. J. Antibiot..

[45] Nett M., König G.M. (2007). The chemistry of gliding bacteria. Nat. Prod. Rep..

[46] Baumann S., Herrmann J., Raju R., Steinmetz H., Mohr K.I., Hüttel S., Harmrolfs K., Stadler M., Müller R. (2014). Cystobactamids: Myxobacterial Topoisomerase Inhibitors Exhibiting Potent Antibacterial Activity. Angew. Chem. Int. Ed..

[47] Surup F., Viehrig K., Mohr K.I., Jansen R., Herrmann J., Müller R. (2014). Disciformycins A and B, unprecedented 12-membered Macrolide-Glycoside Antibiotics from the Myxobacterium Pyxidicoccus fallax active against multiresistant Staphylococci. Angew. Chem. Int. Ed..

[48] Plaza A., Garcia R., Bifulco G., Martinez J.P., Hüttel S., Sasse F., Meyerhans A., Stadler M., Müller R. (2012). Aetheramides A and B, Potent HIV-Inhibitory Depsipeptides from a Myxobacterium of the New Genus “*Aetherobacter*”. Org. Lett..

[49] Gerth K., Bedorf N., Irschik H., Höfle G., Reichenbach H. (1994). The soraphens: A family of novel antifungal compounds from Sorangium cellulosum (Myxobacteria). I. Soraphen A1 alpha: Fermentation, isolation, biological properties. J. Antibiot..

[50] Gerth K., Bedorf N., Höfle G., Irschik H., Reichenbach H. (1996). Epothilons A and B: Antifungal and cytotoxic compounds from *Sorangium cellulosum* (Myxobacteria). Production, physico-chemical and biological properties. J. Antibiot..

[51] Sasse F., Steinmetz H., Schupp T., Petersen F., Memmert K., Hofmann H., Heusser C., Brinkmann V., von Matt P., Höfle G. (2002). Argyrins, immunosuppressive cyclic peptides from myxobacteria. I. Production, isolation, physico-chemical and biological properties. J. Antibiot..

[52] Held J., Gebru T., Kalesse M., Jansen R., Gerth K., Müller R., Mordmüller B. (2014). Antimalarial activity of the myxobacterial macrolide chlorotonil a. Antimicrob. Agents Chemother..

[53] Wenzel S.C., Müller R. (2009). The impact of genomics on the exploitation of the myxobacterial secondary metabolome. Nat. Prod. Rep..

[54] Schneiker S., Perlova O., Kaiser O., Gerth K., Alici A., Altmeyer M.O., Bartels D., Bekel T., Beyer S., Bode E. (2007). Complete genome sequence of the myxobacterium *Sorangium cellulosum*. Nat. Biotechnol..

[55] Goldman B.S., Nierman W.C., Kaiser D., Slater S.C., Durkin A.S., Eisen J.A., Ronning C.M., Barbazuk W.B., Blanchard M., Field C. (2006). Evolution of sensory complexity recorded in a myxobacterial genome. Proc. Natl. Acad. Sci. USA.

[56] Steinmetz H., Mohr K.I., Zander W., Jansen R., Gerth K., Müller R. (2012). Indiacens A and B: Prenyl Indoles from the Myxobacterium *Sandaracinus amylolyticus*. J. Nat. Prod..

[57] Mohr K.I., Garcia R.O., Gerth K., Irschik H., Müller R. (2012). *Sandaracinus amylolyticus* gen. nov., sp. nov., a starch-degrading soil myxobacterium, and description of Sandaracinaceae fam. nov. IJSEM.

[58] Garcia R., Stadler M., Gemperlein K., Müller R. (2015). *Aetherobacter fasciculatus* gen. nov., sp. nov. and *Aetherobacter rufus* gen. nov., sp. nov., two novel myxobacteria with promising biotechnological applications. IJSEM.

[59] Jansen R., Mohr K.I., Bernecker S., Stadler M., Müller R. (2014). Indothiazinone, an indolyl-thiazolyl-ketone from a novel myxobacterium belonging to the Sorangiineae. J. Nat. Prod..

[60] Sood S., Awal R.P., Wink J., Mohr K.I., Rohde M., Stadler M., Kämpfer P., Glaeser S., Schumann P., Garcia R. (2014). *Aggregicoccus edonensis* gen. nov., sp. nov., an unusually aggregating myxobacterium isolated from a soil sample. IJSEM.

[61] Karwehl S., Mohr K.I., Jansen R., Sood S., Bernecker S., Stadler M. (2015). Edonamides, the first secondary metabolites from the recently described Myxobacterium *Aggregicoccus edonensis*. Tetrahedron Lett..

[62] Muyzer G., Bell C.R., Brylinsky M., Johnson-Green P. (2000). Genetic fingerprinting of microbial communities: Present status and future perspective. Microbial Biosystems: New Frontiers, Proceedings of the 8th International Symposium, Halifax, Canada, 9–14 August 1998.

[63] Vaz-Moreira I., Silva M.E., Manaia C.M., Nunes O.C. (2008). Diversity of bacterial isolates from commercial and homemade composts. Microbiol. Ecol..

[64] Winterberg H. (1898). Zur Methodik der Bakterienzahlung. Z. Hyg..

[65] Ward D.M., Weller R., Bateson M.M. (1990). 16S rRNA sequences reveal numerous uncultured microorganisms in a natural community. Nature.

[66] Lewis K. (2013). Platforms for antibiotic discovery. Nat. Rev. Drug Disc..

[67] Lomolino M.V., Riddle B.R., Whittaker R., Brown J.H. (2010). Biogeography.

[68] Ramette A., Tiedje J.M. (2005). Biogeography: An Emerging Cornerstone for Understanding Prokaryotic Diversity, Ecology, and Evolution. Microb. Ecol..

[69] Hanson C.A., Fuhrman J.A., Horner-Devine M.-C., Martiny J.B.H. (2012). Beyond biogeographic patterns: Processes shaping the microbial landscape. Nat. Rev. Microbiol..

[70] DeLong E.E., Pace N.R. (2001). Environmental diversity of Bacteria and Archaea. Syst. Biol..

[71] Bano N., Ruffin S., Ransom B., Hollibaugh J.T. (2004). Phylogenetic composition of Arctic Ocean archaeal assemblages and comparison with Antarctic assemblages. Appl. Environ. Microbiol..

[72] Glöckner F.O., Zaichikov E., Belkova N., Denissova L., Pernthaler J., Pernthaler A., Amann R. (2000). Comparative 16S rRNA analysis of lake bacterioplankton reveals globally distributed phylogenetic clusters including an abundant group of actinobacteria. Appl. Environ. Microbiol..

[73] Rejmánková E., Komárek J., Komárková J. (2004). Cyanobacteria—A neglected component of biodiversity: Patterns of species diversity in inland marshes of northern Belize (Central America). Divers. Distrib..

[74] Hedlund B.P., Staley J.T., Bull A.T. (2003). Microbial endemism and biogeography. Microbial Diversity and Bioprospecting.

[75] Nemergut D.R., Costello E.K., Hamady M., Lozupone C., Jiang L., Schmidt S.K., Fierer N., Townsend A.R., Cleveland C.C., Stanish L. (2011). Global patterns in the biogeography of bacterial taxa. Environ. Microbiol..

[76] Lozupone C.A., Knight R. (2007). Global patterns in bacterial diversity. Proc. Natl. Acad. Sci. USA.

[77] Jiang D.M., Kato C., Zhou X.W., Wu Z.H., Sato T., Li Y.Z. (2010). Phylogeographic separation of marine and soil myxobacteria at high levels of classification. ISME J..

[78] Brinkhoff T., Fischer D., Vollmers J., Voget S., Beardsley C., Thole S., Mussmann M., Kunze B., Wagner-Döbler I., Daniel R. (2012). Biogeography and phylogenetic diversity of a cluster of exclusively marine myxobacteria. IJSEM.

[79] Wielgoss S., Didelot X., Chaudhuri R.R., Liu X., Weedall G.D., Velicer G.J., Vos M. (2016). A barrier to homologous recombination between sympatric strains of the cooperative soil bacterium *Myxococcus xanthus*. ISME J..

[80] Kraemer S.A., Wielgoss S., Fiegna F., Velicer G.J. (2016). The biogeography of kin discrimination across microbial neighbourhoods. Mol. Ecol..

[81] Gerth K., Pradella S., Perlova O., Beyer S., Müller R. (2003). Myxobacteria: Proficient producers of novel natural products with various biological activities—Past and future biotechnological aspects with the focus on the genus Sorangium. J. Biotechnol..

[82] Velicer G.J., Mendes-Soares H., Wielgoss S., Yang Z., Higgs P.I. (2014). Whence Comes Social Diversity? Ecological and Evolutionary Analysis of the Myxobacteria. Myxobacteria: Genomics, Cellular and Molecular Biology.

[83] Tian F., Yong Y., Chen B., Li H., Yao Y.-F., Guo X.-K. (2009). Bacterial, archaeal and eukaryotic diversity in Arctic sediment as revealed by 16S rRNA and 18S rRNA gene clone libraries analysis. Polar Biol..

[84] Wu Z.H., Jiang D.M., Li P., Li Y.Z. (2005). Exploring the diversity of myxobacteria in a soil niche by myxobacteria-specific primers and probes. Environ. Microbiol..

[85] Jiang D.M., Wu Z.H., Zhao J.Y., Li Y.Z. (2007). Fruiting and non-fruiting myxobacteria: A phylogenetic perspective of cultured and uncultured members of this group. Mol. Phylogenet. Evol..

[86] Anderson K.E., Russell J.A., Moreau C.S., Kautz S., Sullam K.E., Hu Y., Basinger U., Mott B.M., Buck N., Wheeler D.E. (2012). Highly similar microbial communities are shared among related and trophically similar ant species. Mol. Ecol..

[87] Dedysh S.N., Pankratov T.A., Belova S.E., Kulichevskaya I.S., Liesack W. (2005). Phylogenetic Analysis and In Situ Identification of Bacteria Community Composition in an Acidic Sphagnum Peat Bog. Appl. Environ. Microbiol..

[88] Hook L.A. (1977). Distribution of Myxobacters in Aquatic Habitats of an Alkaline Bog. AEM.

[89] Rückert G. (1979). Myxobakterien-Artenspektren von Boden in Abhängigkeit von bodenbildenden Faktoren unter besonderer Berücksichtigung der Bodenreaktion. Z. Pflanzenernaehr. Bodenkd..

[90] Pacha R.E., Porter S. (1968). Characteristics of Myxobacteria Isolated from the Surface of Freshwater Fish. Appl. Microbiol..

[91] Li S.G., Zhou X.W., Li P.F., Han K., Li W., Li Z.F., Wu Z.H., Li Y.Z. (2012). The existence and diversity of myxobacteria in lake mud—A previously unexplored myxobacteria habitat. Environ. Microbiol. Rep..

[92] Kou W., Zhang J., Lu X., Ma Y., Mou X., Wu L. (2016). Identification of bacterial communities in sediments of Poyang Lake, the largest freshwater lake in China. SpringerPlus.

[93] Ji Y., Angel R., Klose M., Claus P., Marotta H., Pinho L., Enrich-Prast A., Conrad R. (2016). Structure and function of methanogenic microbial communities in sediments of Amazonian lakes with different water types. Environ. Microbiol..

[94] Kandel P.P., Pasternak Z., van Rijn J., Nahum O., Jurkevitch E. (2014). Abundance, diversity and seasonal dynamics of predatory bacteria in aquaculture zero discharge systems. FEMS Microbiol. Ecol..

[95] Zhang X., Yao Q., Cai Z., Xie X., Zhu H. (2013). Isolation and Identification of Myxobacteria from Saline-Alkaline Soils in Xinjiang, China. PLoS ONE.

[96] Kaushal S.S., Likens G.E., Pace M.L., Utz R.M., Haq S., Gorman J., Gresea M. (2018). Freshwater salinization syndrome on a continental scale. Proc. Natl. Acad. Sci. USA.

[97] Brockman E.R. (1963). Fruiting myxobacteria from the South Carolina coast. J. Bacteriol..

[98] Li B., Yao Q., Zhu H. (2014). Approach to analyze the diversity of myxobacteria in soil by semi-nested PCR-denaturing gradient gel electrophoresis (DGGE) based on taxon-specific gene. PLoS ONE.

[99] Albataineh H.D., Stevens D.C. (2018). Marine Myxobacteria: A Few Good Halophiles. Mar. Drugs.

[100] Schäberle T.F., Goralski E., Neu E., Erol O., Hölzl G., Dörmann P., Bierbaum G., König G.M. (2010). Marine myxobacteria as a source of antibiotics--comparison of physiology, polyketide-type genes and antibiotic production of three new isolates of *Enhygromyxa salina*. Mar. Drugs.

[101] Fudou R., Iizuka T., Sato S., Ando T., Shimba N., Yamanaka S. (2001). Haliangicin, a novel antifungal metabolite produced by a marine myxobacterium. 2. Isolation and structural elucidation. J. Antibiot..

[102] Felder S., Dreisigacker S., Kehraus S., Neu E., Bierbaum G., Wright P.R., Menche D., Schäberle T.F., König G.M. (2013). Salimabromide: Unexpected chemistry from the obligate marine myxobacterium *Enhygromxya salina*. Chemistry.

[103] Felder S., Kehraus S., Neu E., Bierbaum G., Schäberle T.F., König G.M. (2013). Salimyxins and enhygrolides: Antibiotic, sponge-related metabolites from the obligate marine myxobacterium *Enhygromyxa salina*. ChemBioChem.

[104] Sun Y., Tomura T., Sato J., Iizuka T., Fudou R., Ojika M. (2016). Isolation and Biosynthetic Analysis of Haliamide, a New PKS-NRPS Hybrid Metabolite from the Marine Myxobacterium *Haliangium ochraceum*. Molecules.

[105] Treude N., Rosencrantz D., Liesack W., Schnell S. (2003). Strain FAc12, a dissimilatory iron-reducing member of the *Anaeromyxobacter* subgroup of Myxococcales. FEMS Microbiol. Ecol..

[106] Thomas S.H., Padilla-Crespo E., Jardine P.M., Sanford R.A., Löffler F.E. (2009). Diversity and distribution of anaeromyxobacter strains in a uranium-contaminated subsurface environment with a nonuniform groundwater flow. AEM.

[107] Lin J., Ratering S., Schnell S. (2011). Microbial iron cylce in corrosion material of drinking water pipelines. Ann. Agrar. Sci..

[108] Kudo K., Yamaguchi N., Makino T., Ohtsuka T., Kimura K., Dong D.T., Amachi S. (2013). Release of arsenic from soil by a novel dissimilatory arsenatereducing bacterium, *Anaeromyxobacter* sp. strain PSR-1. AEM.

[109] Kim M., Oh H.S., Park S.C., Chun J. (2014). Towards a taxonomic coherence between average nucleotide identity and 16S rRNA gene sequence similarity for species demarcation of prokaryotes. IJSEM.

[110] Zerzghi H., Brooks J.P., Gerba C.P. (2010). Pepper IL. Influence of long-term land application of Class B biosolids on soil bacterial diversity. J. Appl. Microbiol..

[111] Mohr K.I., Moradi A., Glaeser S.P., Kämpfer P., Gemperlein K., Nübel U., Schumann P., Müller R., Wink J. (2018). *Nannocystis konarekensis* sp. nov., a novel myxobacterium from an Iranian desert. IJSEM.

[112] Xu K., Liu H., Li X., Chen J., Wang A. (2010). Typical methanogenic inhibitors can considerably alter bacterial populations and affect the interaction between fatty acid degraders and homoacetogens. Appl. Microbiol. Biotechnol..

[113] Hansel C.M., Fendorf S., Jardine P.M., Francis C.A. (2008). Changes in bacterial and archaeal community structure and functional diversity along a geochemically variable soil profile. Appl. Environ. Microbiol..

[114] Brockman E.R. (1976). Myxobacters from Arid Mexican Soil. AEM.

[115] Gerth K., Müller R. (2005). Moderately thermophilic Myxobacteria: Novel potential for the production of natural products isolation and characterization. Environ. Microbiol..

[116] Iizuka T., Tokura M., Jojima Y., Hiraishi A., Yamanaka S., Fudou R. (2006). Enrichment and Phylogenetic Analysis of Moderately Thermophilic Myxobacteria from Hot Springs in Japan. Microbes Environ..

[117] Dawid W., Gallikowski C.A., Hirsch P. (1988). Psychrophilic myxobacteria from Antarctic soils. Polarforschung.

[118] Brockman E.R., Boyd W.L. (1963). Myxobacteria from soils of the Alaskan and Canadian arctic. J. Bacteriol.

[119] Müller R., Wink J. (2014). Future potential for anti-infectives from bacteria—How to exploit biodiversity and genomic potential. Int. J. Med. Microbiol..

